# Histological Studies of Mycorrhized Roots and Mycorrhizal-Like-Structures in Pine Roots

**DOI:** 10.3390/mps1030034

**Published:** 2018-09-05

**Authors:** Carla Ragonezi, Maria Amely Zavattieri

**Affiliations:** 1 Banco de Germoplasma ISOPlexis, Campus da Penteada, Universidade da Madeira, 9020-105 Funchal, Portugal; zavattieri@uevora.pt; 2 Departamento de Biologia, Pólo da Mitra Apartado 94, 7002-554 Évora, Portugal; 3 Instituto de Ciências da Terra (ICT), Colégio Luís António Verney, Rua Romão Ramalho 59, 7000-671 Évora, Portugal

**Keywords:** ectomycorrhiza, mycorrhiza-like structures, stone pine, adventitious roots, Hartig net

## Abstract

Several studies have shown the potential of using Ectomycorrhizal (ECM) fungi in conifer micropropagation to overcome the cessation of adventitious root development. In vitro inoculation promotes the re-growth of the root system induced previously by auxin treatments, facilitating acclimation and diminishing the losses of plants because of a weak root system that is incapable of water and nutrient absorption. During a series of mycorrhization experiments, cryostat and ultrafine cuts were used to study the morpho-histological transformation of the symbiotic roots. To obtain cryostat cuts from pine roots a method frequently used for animal tissue was adopted. Molecular methods allowed fungi identification in all the mycorrhization phases and in the acclimation of derived plants. Mycorrhizal-like-structures derived from in vitro culture and axenic liquid cultures of roots were microscopically analyzed and compare with mycorrhizal roots.

## 1. Introduction 

Ectomycorrhizal fungi (ECM fungi) are phylogenetically very diverse and more than 2000 species of ECM fungi worldwide have been identified, primarily from *Basidiomycota* and *Ascomycota*. ECM fungi are associated mainly with woody perennials, including *Pinaceae*, *Betulaceae*, *Fagaceae*, and *Diperocarpaceae* in tropical, subtropical, and arid environments, and are regarded as key organisms in nutrient and carbon cycles in forest ecosystems [1]. Most fine roots and ECM fungi of trees are aggregated in the uppermost 20 cm of soil where nutrient circulation is high [2]. Ectomycorrhizas are characterized primarily by the presence of a sheath or a mantle of fungal tissue around the root, the Hartig-net, consisting of modified fungal hyphae that develops between root cells and a long system of fungal hyphae that connects the soil with the fruit bodies of the fungi forming the ectomycorrhizas [3,4]. This relates not only to the extent of root colonization but also to the development of hyphae in the soil. This mutualistic relationship with ECM fungi grants conifers an ecological advantage to withstand harsh living conditions. The way to recognize an ectomycorrhiza is through the structural modification of roots (dichotomous, coralloid or short monopodial roots) that are totally different from non-mycorrhized roots [5]. Another way to recognize them is via histological analysis in which the fungal mantle and Hartig-net are observed. Recently, molecular techniques display the possibilities to identify, by gene expression, the presence of mycorrhizas. Antibodies and cDNA probes of the genes *PF6.2* and LbRas of the mycorrhiza *Laccaria bicolor*, were also expressed in several other fungi that form mycorrhiza with red pine (*Pinus resinosa*) [6].

Biotechnological research for the clonal propagation of stone pine (*Pinus pinea* L.) was done previously in Portuguese research, since this important Mediterranean pine constitutes a relevant resource for the Portuguese economy mostly due to its edible seeds. After obtaining in vitro microshoots from mature cotyledons, it was possible to induce roots by the addition of growth regulators in the culture medium and modifying some physical parameters [7,8], but after the expression phase roots stop growing. This fact reduces the possibilities for acclimation of the micro pine plants and the clonal production within a commercial scale. To overcome this limitation a co-culture system with ectomycorrhizal fungi was developed [9]. The successful co-culture system methodology between stone pine plants with several ectomycorrhizal fungi belonging to the genus *Lactarius, Pisolithus*, *Laccaria* and *Hebeloma*, was patented under the Portuguese patent number PT 105239 [10].

To evaluate and validate the efficiency of the co-culture system, visual examination of the morphological changes of the root system was made during the acclimation phase in mixed substrate and in Rhizotrons, which allowed the direct observation of modified pine roots by different fungi. However, direct observation was sometimes not enough even for a trained researcher and thus microscopic analysis of the root was necessary. The morphological modifications of the root structure include monopodial, dichotomous, and/or coralloid branching of lateral roots; inhibition of root hair formation; and enlargement of cortical cells. 

During in vitro rooting, mycorrhizal-like structures, very similar to EMC colonized roots, appeared in some of the inoculated clones. These root modifications, very similar to mycorrhizas, appeared preferentially in jars left for a long time without any new media transference, and therefore in a situation of stress, possibly water stress due to culture media desiccation. These mycorrhiza-like structures were reported before in nature in *Pinus pinaster* [11], *Pinus mugo* [12], *Pinus taeda, Pinus muricata*, and *Pinus halepensis* [13]. Different pine species show different abilities to form mycorrhiza-like structures. *Pinus pinea* is not the exception.

The objective of this paper is to focus on protocols and methods followed or adapted from other protocols to study the morpho-anatomical root changes derived from ectomycorrhizal inoculation and mycorrhizal-like-structures. Evaluation of the presence or absence of ectomycorrhizas in the modified roots of *P. pinea* was possible by using ultra-microtome cuts and cryostat micro cuts. The cryostat samples of the roots were obtain preparing the tissue according to a methodology generally used for preparation of animal tissue samples as described in material and methods. 

## 2. Material and Methods

### 2.1. Plant Material 

Mature seeds of stone pine were obtained from selected plus trees (Alcácer do Sal, Portugal) and were stored in a cold chamber at 4 °C until used. For the details of in vitro shoot induction from cotyledons of mature seeds and shoot multiplication see [14]. Microshoots were placed in rooting medium WPMRI (Wood Plant Medium root induction) and after, in WPMRE (Wood Plant Medium root expression) [15].

### 2.2. Fungi

Sporocarps of several ectomycorrhizal fungi including Pisolithus arhizus (Scop.) Rauschert, Hebeloma cylindrosporum Romagn., Russula torulosa Bres., Lactarius deliciosus (L.) Gray., Rhizopogon luteolus Fr., Laccaria laccata (Scop.) Cooke, Suillus bellinii (Inzenga) Kuntze were collected from a pure stand of stone pine in Portugal (N 38°25′; W 7°56′) in winter after a few days of heavy rainfall. In situ was done the preliminary identification via morphological traits and specimens were stored at 4 °C prior to sterilization and isolation procedures. 

### 2.3. Mycelia Isolation and Fungal Cultures

**Figure 1:**
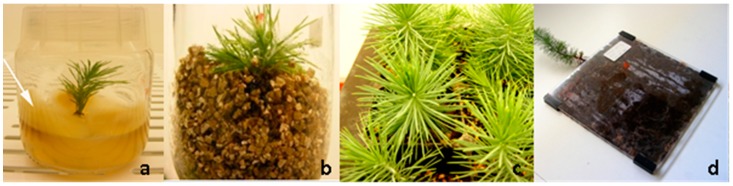
In vitro co-culture system between stone pine microshoots with some developed roots induced by 2-Naphthaleneacetic acid (NAA) and *Pisolithus* sp. (white arrow) in the surface of the double layer medium (**a**). Acclimation of mycorrhized pine plants in vermiculite (**b**) and in mixed substrates (**c**) from which root samples were taken for cryostat analysis. Some mycorrhized plants were transferred to rhizotrons for direct observation of mycorrhizal morphological structures (**d**).

The methodology used was according to [16], briefly, the fruiting bodies (sporocarps) were cut into pieces and disinfected by placed in running water for 10 min and then in 70% ethanol for 2 min. Then, pieces were rinsed with sterile distilled water in a laminar flow chamber, placed in 20% (*v*/*v*) commercial bleach (≤5% active chlorine) for 10 min and rinsed four times with sterile water. Larger pieces were then cut in smaller pieces (50 mm^3^) for growth. The isolation and culture of the ECM fungi species was made in modified Hagen medium [17]. The formulation of modified Hagen (per liter of medium) was: KH_2_PO_4_ 0.5 g, NH_4_CL 0.5 g, MgSO_4_·7H_2_O 0.5 g, FeCL_3_ (1%) 0.5 mL, glucose 5 g, malt extract 5 g, thiamine HCL 50 μg, and agar 15 g. The pH was adjusted to 4.5–5.0. The media was autoclaved for 20 min at 121 °C, 1 atm, and 100 mg mL^−1^ of Rifampicin (Sigma-Aldrich^®^, Saint Louis, MO, USA) was added to the media after cooling to avoid the contamination by bacteria. Pieces of sporocarps in this medium were kept at 25 °C in the dark and subculture at weekly intervals. After 14 days in culture the mycelia were used for co-cultures (Figure 1a). 

### 2.4. In Vitro Co-Culture

Following the in vitro rooting induction and expression phase, pine roots generally stop the growth, this is an evidence of recalcitrance in this species and the main reason to use mycorrhizal inoculation. In a study by Oliveira et al. [14], it was demonstrated that the co-culture system was extremely useful to cope with the difficulties of rooting. Microshoots were transferred to the double-layer medium and, after a brief period of adaptation into the medium, inoculated with selected fungi. Control plants were also transferred to a double-layer medium but were not inoculated. 

### 2.5. Acclimation 

After the co-culture, plants of all treatments including control plants (without mycorrhization) went through acclimation with the aim to follow the ex vitro growth of the root system. The plantlets were first transferred to sterile vermiculite for two weeks and then transferred to a mixed substrate of vermiculite/perlite/peat in a proportion of 2:1:1. The acclimation lasted 10 weeks in a growth chamber at 25/19 °C day/night temperatures, with 16 h photoperiod (270 μmol m^−2^ s^−1^) and a relative humidity of 80%. Glass jars were covered with plastic film in which some holes were made to ensure gas exchange and to ensure a rapid acclimation. A high 80% relative humidity was maintained in the growth chamber, during the first week and was gradually decreased to 60%. Plants were watered as required, alternating between sterile water and liquid WPM (macronutrients only). 

### 2.6. Rhizotron

In most of the experiment, some clonal plants inoculated with ectomycorrhizal fungi *Pisolithus arhizus* (Scop.) Rauschert and *Lactarius deliciosus* (L. ex Fr.) SF Gray, were transferred to rhizotrons. Rhizotrons allow the visualization of root development and changes in the appearance of the root derived from different fungi colonization, whenever desired without disturbing the normal functions of the plants. Basically, they are made of two acrylic plates, 20 × 20 cm each, with intervals made by 5 mm spacers and filled with *Sphagnum* sterilized turf, to support and feed the plants [18], with some adaptations later proposed [19]. Observation of the roots was performed from 4 to 6 weeks, which is the period required for most plants to explore the available space. 

### 2.7. Cryostat 

Mycorrhized root samples from the acclimation to the mixed substrates were collected at the end of each experiment and identified for histological and anatomical studies. Roots were fixed in 4% glutaraldehyde, diluted in 0.1 M HEPES buffer 2-[4-(2-hydroxyethyl) piperazin-1-yl] ethanesulfonic acid, pH 6.8 and stored in a refrigerator (at 4°) for 24 h. Afterwards, roots were washed twice in 1N PBS phosphate buffer saline (PBS) with 4% sucrose for 15 min and finally washed in PBS with 15% sucrose. After washing, roots were placed in PBS with 15% sucrose and 7.5% of microbiology gelatin (Merck^®^, Lisbon, Portugal) for 1 to 2 h at 37 °C. Other Petri dishes with the same gelatin base were prepared and, after 1 h when the solution solidified, roots were placed on the surface of the gelatin and covered with a 1 cm layer of molten gelatin solution. Petri dishes were stored in a refrigerator for 1 h; blocks (1 cm × 1 cm × 1 cm) of gelatin with roots were cut out, frozen, and stored at −80 °C. Longitudinal and transverse sections, approximately 5–10 μm thick, were cut at −33 °C using a cryostat Leica CM3050 S (Leica Biosystems, Wetzlar, Germany), transferred to glass slides and stained with a common fountain pen ink. Sections were observed under an Olympus (Southend-on-Sea, UK) microscope at a magnification of 1125×.

### 2.8. Solution Gelatin 

For a 100 mL solution, gradually mix 7.5 g of microbiology gelatin (Merck^®^) powder with PBS (can be heated up to 55 °C) to go into solution. Add 15 g of sucrose and mix until dissolved (you can warm up to 55 °C to help). Set the volume with PBS and place at 37 °C in a hot air oven. Equilibrate the temperature for 30 min before placing the tissue in the gelatin.

### 2.9. Axenic Root Cultures and Microscopic Analysis 

To induce the formation of mycorrhizas-like-structures for comparison with symbiotic ectomycorrhizal structures the methodology used was according to [20], briefly, root segments of 2 cm long, obtained from germinated pine seeds (pinions), were excised and cultured in liquid medium in an orbital shaker (125 rpm) for 3 to 4 weeks. Afterward, the roots were photographed and used for histological studies. Two different methods were used (1) hand sections of root segments were obtained, segments were placed between pieces of laboratory Parafilm and cut as thin as possible using a razor blade [16] and observed under a Zoom Stereo Research Microscope 7-70X Olympus SZH10. Structural details of the root anatomy were observed under a Light Microscope with image acquisition device Olympus CX-40 and photographs were taken with a Canon (Tokio, Japan) Power-Shot A630 camera. (2) Dichotomous and short roots were fixed in FAA (dehydrated in an aqueous series of ethanol (70, 80, 95, 100%), clarified in xylol, embedded in paraffin, and cut with a rotary microtome (8–10 μm). The sections were stained with toluidine blue, mounted in Entellan^®^ (rapid mountain medium for microscopy) and observed under a Light Microscope [21]. These methods were also used in mycorrhized roots as a complement of the previous described using a cryostat cuts and stain.

### 2.10. Identification of the Fungal Isolates

The identification of the fungi was first made at the site of the collection of the sporocarps in the stands of pine. This characterization was based on their morphotypes. To confirm the identity of the collected material, internal transcribed spacer (ITS) amplification of the ribosomal genes was applied using the pure cultures derived from the sporocarps. Small subunit 18S, and the 5.8S of the rDNA repeat unit was amplified using the oligonucleotides primers ITS 5 and ITS4. Polymerase chain reaction (PCR) products were analyzed by gel electrophoresis and sequenced by capillary electrophoresis. The sequences of the ITS region were aligned with related fungal strains from the GenBank databases for the homology analysis. Also, M13-PCR fingerprinting methodology for monitoring different species of *Basidiomycota* and *Ascomycota* associated with stone pine was tested. For details about these methods used see Ragonezi et al. [22].

## 3. Results 

**Figure 2:**
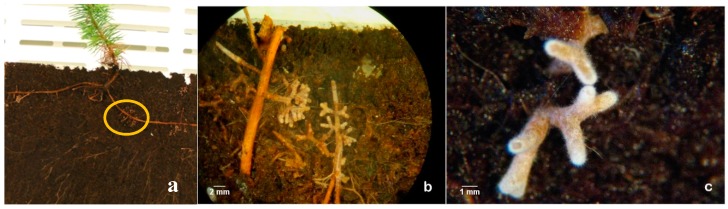
Pine plant in the rhizotron (**a**) where it is easy to observe mycorrhizas and hyphae in the turf substrate. Turf maintains an elevated humidity that favors the growth of fungi. Magnification of mycorrhizas in a stereomicroscopy, 10×, scale bar 2 mm (**b**). Detail of dichotomous branching and hairy white hyphae 50x, scale bar 1 mm (**c**).

**Figure 3:**
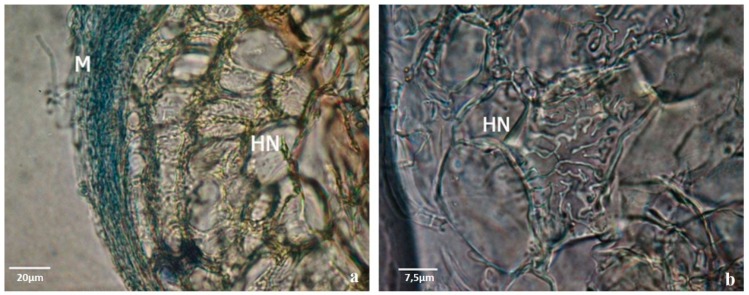
Cryostat transversal section of pine root colonized by *Pisolithus* sp. showing the mantle hyphae around the root surface (M) (200×), scale bar 20 µm (**a**). Internal Hartig-net (HN). Details of the transverse section showing well-differentiated Hartig-net (HN) in cortical cells of the root (2000×). Scale bar 7.5 µm (**b**).

All the ECM fungi tested enhance rooting as it was expected, but the number of new roots, and the length of them varied between ECM fungi tested. Higher number of roots and branches obtained by the presence of the mycorrhizal fungi was possible because ECM fungi exude (among many other chemical compounds) auxins, that directly influence the root growth. Re-growth and the production of new roots and branches is highly advantageous during acclimation phase of pine plants. Among the ECM tested, *Pisolithus arhizus* was selected here to exemplify our results. Later it was also used in the experiments on biochemical signaling with microplants of stone pine [9]. The co-culture of *P. pinea* microshoots with *Pisolithus* sp. (Figure 1a) effectively helped to overcome one of the most common problems associated with in vitro rooting: the inhibition of root elongation under the culture conditions and the adaptation of the plantlets to ex vitro conditions. Even when an extremely poor substrate like vermiculite was used, during the early phase of acclimation (Figure 1b) none of the inoculated plantlets died and a vast mycorrhizal symbiosis establishment was observed in mixed substrates (Figure 1c) and in rhizotrons (Figure 1d and Figure 2). Moreover, fewer roots were lost during transplantation to mixed substrates which was facilitated by the morphological modifications of the mycorrhized roots such as the presence of the hyphae around the roots (mantle) (Figure 2c and Figure 3) and the internal Hartig-net (Figure 3a), which increased root thickness and contributed to a more robust root system [16].

**Figure 4:**
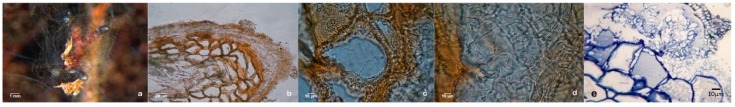
Morphology of the ectomycorrhiza produced between the pine clone plant Fp2 inoculated with the fungus 1N(1)b, scale bar 1 mm (**a**); longitudinal cryostat Section 5 mm (400×), with mantle development scale bar 20 µm (**b**); detail of the mantle hyphae, 1000×, scale bar 10 µm (**c**); Hartig net and fungi between cell walls, 1000×, scale bar 10 µm (**d**) mantle and Hartig net in transverse section obtained with an ultra-microtome (1 µm) stained with Toluidine blue, scale bar 10 µm (**e**).

**Figure 5:**
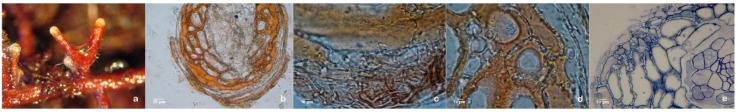
Enlarge images of the ectomycorrhiza produced between pine clone plant Fo2 inoculated with fungus 1M (2)a, scale bar 1 mm (**a**); transverse sec (5 mm) cryostat cut at 400× magnification in microscope, scale bar 20 µm (**b**); mantle hyphae 1000×, scale bar 10 µm (**c**); Hartig net detail 1000×, scale bar 10 µm (**d**) mantle and Hartig-net observed in transverse sections using an ultra-microtome (1), scale bar 10 µm (**e**).

Samples of dichotomous and coralloid roots from the plants grown in mixed substrate and in rhizotrons were collected after 10 weeks in acclimation for a detailed histological study. According to our previous experience, the symbiotic structures were highly variable in their complexity, but the transverse sections of the ectomycorrhizal roots showed a well-developed mantle and Hartig-net for almost all ectomycorrhizal fungi tested (Figure 3a,b, Figure 4 and Figure 5).

Ectomycorrhizal structures produced between pine plants and fungi during the acclimation phase (derived from previous co-cultures) were analyzed by cryostat and ultramicrotome cuts. The ectomycorrhizas were morphologically very different from each other in the dichotomous structure color, hyphal extension and color of the mantle, and in the internal development of the Hartig net. In Figure 4 and Figure 5 it is possible to observe two examples.

**Figure 6:**
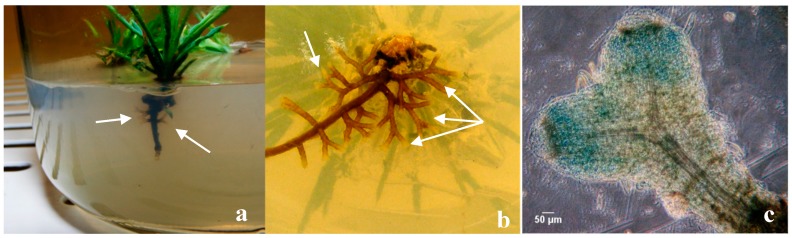
In vitro dichotomous branching of roots (arrows) with no fungi culture in the upper layer medium (**a**). Dichotomous and coralloid branching (arrows) in plants that remain for a long-time in vitro rooting expression medium (**b**) and histological preparation of a dichotomous root without mantle or Hartig-net, scale bar 50 µm (**c**).

Extensive dichotomous and coralloid branching of lateral roots occurred during in vitro rooting at the expression phase in control plants (Figure 6a). Somehow, the question of whether some mycelium could have contaminated the control plants was raised, even thought this was highly improbable. Also, non-inoculated plants that remained in the culture medium for longer than a month, in a drier medium, developed numerous mycorrhizal-like structures (Figure 6b). This would suggest a correlation between osmotic and/or nutritional stress and the abundance of these mimicking structures. The hand-made sections observation confirmed the absence of a mantle and Hartig-net in this ectomycorrhizal-like-structures (Figure 6c) and other important internal and external microscopic differences was found comparing this structure with dichotomous branching roots derived from ectomycorrhizas. 

**Figure 7:**
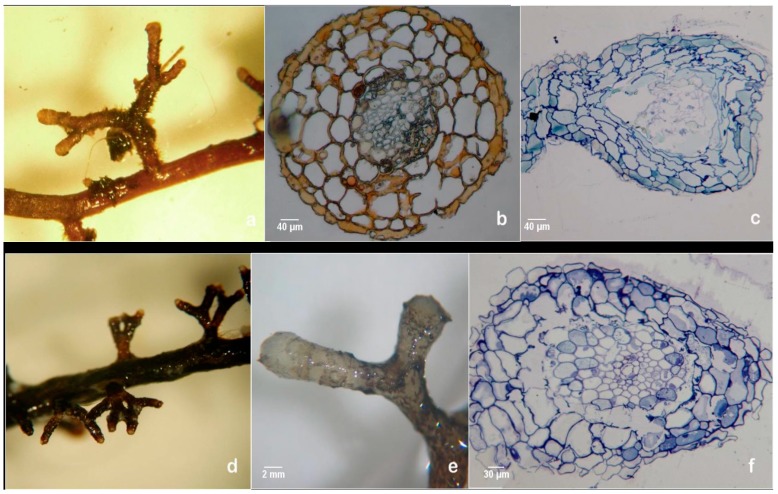
Dichotomous branching in liquid axenic culture clones 122 (**a**) and clone 138 (**d**). Hand-made root cut using parafilm foil; the dichotomous structure did not have a mantle or a Hartig-net, scale bar 40 µm (**b**). Dichotomous roots derived from axenic culture, without mantle, or hyphae around it, scale bar 2 mm (**e**). Ultramicrotome cuts of dichotomous and coralloid roots stained with toluidine blue, no mantle, nor Hartig-net was observed, scale bar 40 µm and scale bar 30 µm (**c** and **f**).

The results in axenic liquid cultures showed that all pine clones tested can produce mycorrhiza-like-structures (Figure 7a,d). Supplementary, to exclude any possibility of the liquid cultures contamination with fungal mycelium, several histological studies were carried out, like hand-made cuts between parafilm foil (Figure 7b) and ultra-microtomy cuts and subsequent staining as shown in Figure 7c,f.

### Species Identification of P. arhizus and L. deliciosus

PCR products of ITS4/ITS5 primers, were 644 bp and 400 bp obtained from dikariontic isolates from *Pisolithus* sp. P1001 and *Lactarius deliciosus* UEZB1, respectively. Sequence alignments of *P. arhizus* showed identities that ranged from 99–100% among isolates belonging to *P. arhizus*; for *L. deliciosus* the homology was over 99%. Both sequences were published in GenBank with accession number HQ896485 and JQ066791, respectively [23,24]. Results demonstrated that M13-PCR discriminated between species and taxonomic groups. Based on the specific PCR fingerprints and the high interspecies variation of the banding patterns, a clear distinction among all species used for the test was viable. M13-PCR highlighted differentiation at the species and strain level [25].

## 4. Discussion 

Apparently, there was a strong morphological similarity between extensive dichotomous and coralloid branching of lateral roots that grew because of changes in the osmotic potential or nutrient content of the culture medium and those derived from fungal inoculation of pine plants. Due to this macroscopic similarity, it may be difficult to diagnose ectomycorrhizas without confirmation either by molecular analysis or by the ECM fungi status via histological analysis. The combination of sequence analysis of the ITS regions of the rDNA and the PCR fingerprinting technique was extremely helpful to identify the species collected in the field as well as to monitor the fungus involved in all the steps in our mycorrhization programs. M13-PCR is a rapid method for DNA amplification of polymorphic sequences. It was possible with M13 to characterize the genetic profile of sporocarps collected such as *L. deliciosus*, *P. arhizus*, and *R. roseolus*. The advantages of this DNA amplification method are simplicity, high levels of resolution, universal availability of the primers, reproducibility, easy database analysis, and the reduced costs [26]. PCR fingerprinting is very helpful to resolve taxonomic problems and to differentiate species and strains of filamentous fungi [27].

The presence of mycorrhiza-like-structures has been previously observed in other pine species and might be indicative of long coevolution of these two kingdoms for millions of years [13,28]. The presence of these structures was reported in other in vitro culture systems between ECM and conifers, for example, *Picea abies* cell culture induced ectomycorrhizal fungi to form mycorrhiza-like structures which normally are only generated in the presence of host roots [29]. It is unclear what is the natural compound associated with the change in root architecture, like that which occurs with strigolactones in the interaction promotion with arbuscular mycorrhizal fungi [30]. 

Histological observations allowed us to definitively separate structures derived from the symbiosis by the fungi of other similar structures induced by other factors (genetics; environmental). The adaptation of a protocol used for animal samples preparation showed to be simple and highly effective to be used for cryostat cuts. In this paper cuts made with the *Pisolithus arhizus* were shown. However, all the ECM fungi tested improved rooting of stone pine and microscopic observations confirmed, for all of them, a good development of the mantle and Hartig-net. 

## 5. Conclusions

The micropropagation of most conifers is often limited by the difficulty of rooting, and it was therefore essential to carry out an extensive bibliographic search on the subject before in vitro culture and biotization experiments. Based on information obtained from various sources, several physical and chemical factors were adjusted to improve in vitro rooting of *P. pinea* microshoots. An improvement in shoot rooting up to 70% was achieved for most of the tested clones (data published elsewhere). However, the roots obtained ceased growth. A new strategy was developed based on the co-cultivation of *P. pinea* and different ectomycorrhizal fungi (biotization). The methodology adopted was presented in this article. Biotization produced positive results, since they effectively contributed to overcoming the cessation of root growth and the improvement of various rooting parameters [9].

In some cases, regenerated adventitious roots may develop mycorrhizal-like structures without the presence of fungus. There was a strong similarity between extensive dichotomous and coralloid branching of lateral roots that grew spontaneously in stone pine with those derived from fungal inoculation. Due to this similarity it may be difficult to diagnose ectomycorrhizas without confirmation of the ECM status by histological analysis. The appearance of mycorrhizal-like-structures depends on environmental conditions, the genetics of the species and the use of growth regulators. Abundant mycorrhizal-like structures in stone pine roots were produced by axenic cultures, in in vitro cultures, and in the subsequent acclimation phase in mixed substrates. More studies will be needed to elucidate the biochemical mediators that modify root architecture and its relationship with other compounds.

Histological studies in combination with molecular techniques (the use of PCR-M13), allowed us to separate the different groups of fungi that can be found in stone pine forests in southern Portugal. In addition, it was possible to monitor mycorrhization stages.
